# Cardiac AL amyloidosis in a veteran endurance athlete with pre-existing apical hypertrophic cardiomyopathy: a case report

**DOI:** 10.1093/ehjcr/ytaf443

**Published:** 2025-09-06

**Authors:** Emmanuel Androulakis, Szymon Musiol, Michael Papadakis, Maria Teresa Tome Esteban

**Affiliations:** Kings College Hospital NHS Foundation Trust, Cardiac Unit, Denmark Hill, London SE5 9RS, UK; King's College London, School of Biomedical Engineering & Imaging, London WC2R 2LS, UK; City St George's, University of London, Cranmer Terrace, London SW17 0RE, UK; Royal United Hospitals NHS Foundation Trust, Cardiology Department, Combe Park, Bath BA1 3NG, UK; St George's University Hospitals NHS Foundation Trust, Cardiology Clinical Academic Group, Blackshaw Road, London SW17 0QT, UK; St George's University Hospitals NHS Foundation Trust, Cardiology Clinical Academic Group, Blackshaw Road, London SW17 0QT, UK

**Keywords:** Hypertrophic cardiomyopathy, Cardiac amyloidosis, Multimodality imaging, Endurance athlete, Case report

## Abstract

**Background:**

Patients with mild phenotypes of chronic cardiomyopathies are often followed up over time, and there is a temptation to assume a change in symptom status or investigation results is due to the original pathology. The possibility of acquired pathology must be considered in these patients.

**Case summary:**

We present a case of a middle-aged veteran endurance athlete followed up for a mild phenotype of apical hypertrophic cardiomyopathy originally picked up due to an asymptomatic abnormal electrocardiogram (ECG). He developed subtle symptoms and morphological changes in his investigations (ECG, echocardiography, exercise tolerance test (ETT), and magnetic resonance imaging (MRI)). Due to the previously quiescent phenotype, a diagnosis of coexistent amyloid light-chain (AL) cardiac amyloidosis secondary to IgA lambda multiple myeloma was made.

**Discussion:**

This case underscores the importance of paying attention to subtle changes in symptoms and morphology when managing patients with mild phenotypes and athletes, and considering alternative pathology when these occur. Multimodality imaging is essential in the investigation of such patients.

Learning points

- New symptoms should not automatically be attributed to progression of the phenotype: co-existent dual pathology should always be considered
- Multimodality imaging is of utmost importance in distinguishing co-existing pathology like amyloidosis from simple progression of a primary cardiomyopathy
- Longitudinal surveillance in patients who exercise requires expert’s assessment taking also into account longitudinal ECG changes, arrhythmia assessment, and performance during exercise

## Introduction

Cardiac muscle problems can present in similar ways regardless of the underlying disease. In patients with cardiomyopathy, there is a temptation to ascribe changes in symptoms to the primary pathology. Our case illustrates how clinical vigilance and multimodality imaging assessment lead to the diagnosis of an acquired heart condition on a background of hypertrophic cardiomyopathy.

## Summary figure

Panels A1 and B1 show evolution of repolarization abnormalities on the 12-lead electrocardiogram between baseline (2015) and presentation (2023). The first ECG shows T-wave inversion in Leads II, III, and aVF, a finding always considered pathological. The J-point elevation in the anterior leads and voltage criteria for LV hypertrophy may represent a degree of athletic remodelling. The second ECG shows superadded T-wave inversion in the lateral leads with associated ST-depression clearly suspicious of pathology. Also, smaller QRS complexes are noted on the second ECG, which could be in keeping with cardiac amyloid.

Panels A2–A4 show the multimodality imaging aspects of the baseline phenotype depicting loss of apical tapering and very mild relative apical hypertrophy, whilst panels B2–B4 show the progression of the phenotype at the time of the amyloid diagnosis. In panel B, very prominent concentric-predominantly mid-apical hypertrophy was noted, alongside a mild pericardial effusion, and on LGE imaging, diffuse subendocardial to mid-wall enhancement was noted in the LV walls and the left atrium. The aforementioned features are key diagnostic elements of cardiac involvement. A2 and B2, apical four-chamber view on transthoracic echocardiography; A3 and B3, apical four-chamber view on cine sequence of cardiac MRI; A4, apical three-chamber view of late gadolinium sequence on MRI; B4, apical three-chamber view of late gadolinium sequence on MRI; LV, left ventricle, RV, right ventricle; LA, left atrium; RA, right atrium; Ao, aortic root.

**Figure:**
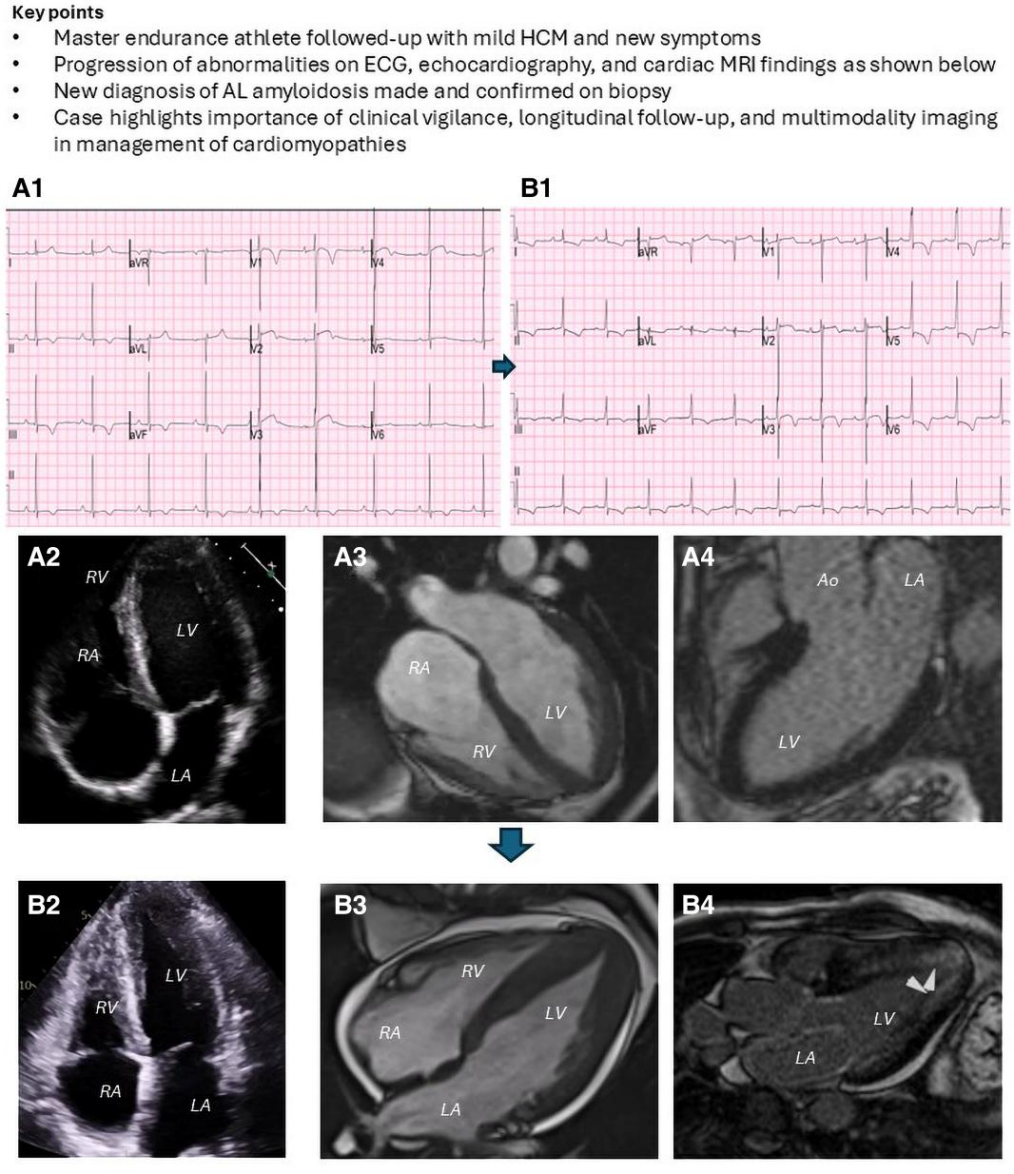


## Case presentation

A 55-year-old veteran marathon runner was under surveillance from the Inherited Cardiac Conditions service with a likely diagnosis of mild apical hypertrophic cardiomyopathy (HCM).^1^ He was originally referred due to an incidental finding of an abnormal ECG (*Summary Figure, panel A1*), showing inferior T-wave inversion, which cannot be attributed to athletic adaptation. The patient had no other past medical history and specifically no hypertension and normal coronary arteries on computed tomography (CTCA). He was a non-smoker and drank no alcohol. His two adult children had been screened and remained well. Genetic testing identified a variant of unknown significance (VUS) in the MYBPC3 gene: c.3343G>A; p.(Val1115Ile), which in itself is not diagnostic or directly supportive of pathology.^2^ Due to the low-risk phenotype, he was reviewed on a 1–2 yearly basis and continued participating in vigorous aerobic exercise: three to four runs of 5–10 km weekly, as well as one long run exceeding 10 km. He regularly participated in several half-marathons and marathons every year. He remained asymptomatic until around a year ago.

In 2023, he presented with a gradual decline in athletic performance and exertional breathlessness with occasional chest tightness for over 1 year. On examination, there was no evidence of heart failure, and the blood pressure was 100/64 mmHg with a regular heart rate of 68 beats per minute. The clinic ECG (*Summary Figure, panel B1*) showed progression of repolarization abnormalities, and the ST-depression was a new finding (*Table 1*). The most recent ECG showed superadded T-wave inversion in the lateral leads with associated ST-depression clearly suspicious of pathology. Also, smaller QRS complexes were noted on the second ECG, which could be in keeping with cardiac amyloid. His laboratory results are summarized in *Table 2*. The patient did not perform strenuous exercise around the time of testing.

**Table 1:** Summary of laboratory investigations

Parameter	Value	Unit	Reference range
Haemoglobin	147	g/L	130–180
White cell count	4.9	10^9^/L	3.6–11.0
Platelets	156	10^9^/L	140–400
Creatinine	91	μmol/L	59–104
NT-pro-BNP	3154	ng/L	<125
Troponin T	46	ng/L	<14
IgA	9	g/L	0.8–3.0
Kappa free light chains	13.5	mg/L	3.3–19.4
Lambda free light chains	177.33	mg/L	5.7–26.3^Abbreviations^
Kappa/lambda ratio	0.08	–	0.26–1.65

NT-proBNP, N-terminal prohormone of Brain Natriuretic Peptide.

**Table 2:** Comparison between baseline characteristics and parameters at the time of AL amyloid diagnosis

	Baseline	October 2023	Unit
12-lead electrocardiogram			
PR interval	218	229	ms
QRS duration	88	94	ms
Corrected QT interval (QTc)	457	479	ms
T-wave inversion	II, III, aVF	II, III, aVF, V3, V4, V5, V6, I, aVL	
ST-segment depression	–	V4, V5, V6	
Echocardiogram			
LV maximum wall thickness	11.5	18	mm
IVSd	11.5	14	mm
LV EF	Visually normal	65	%
*E*/*e′*	5.5	15	
RV free wall thickness	N/A	11	mm
GLS	N/A	−6.2	%
Estimated PASP		23–28	mmHg
Cardiac MRI			
LV mass index	90	119	g/m^2^
Native T1 time	1350	1084	ms
ECV		44	%

LV, left ventricle; RV, right ventricle; GLS, global longitudinal strain; PASP, pulmonary artery systolic pressure; ECV, extracellular volume.

**Figure 1:**
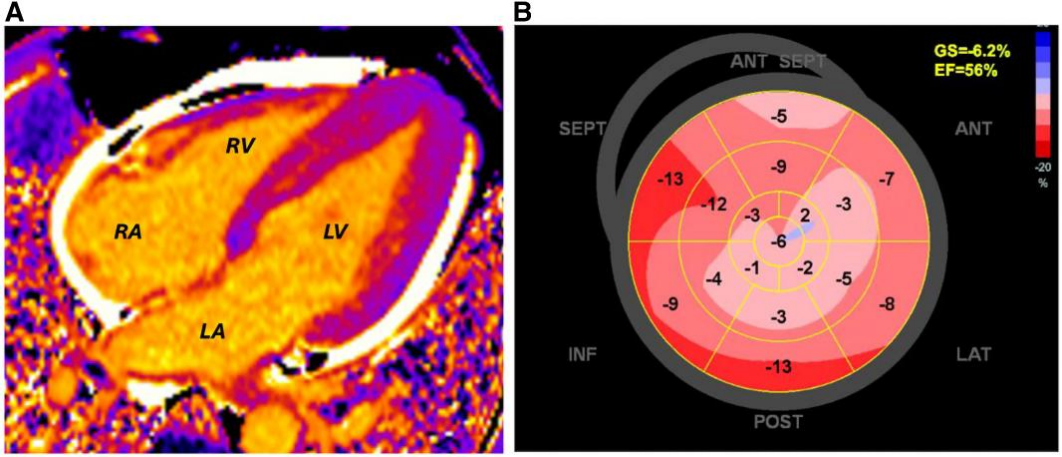
(*A*) Native T1 mapping of 2023 MRI (apical four-chamber view). Purple areas correspond to lower values and orange areas to higher values. The overall T1 time was 1084 ms. (*B*) Longitudinal strain map of 2023 echocardiogram. LV, left ventricle; RV, right ventricle; LA, left atrium; RA, right atrium; GS, global strain; EF, ejection fraction; ANT SEPT, anteroseptal; SEPT, septal; INF, inferior; POST, posterior; LAT, lateral; ANT, anterior.

In-clinic echocardiogram in 2023 revealed prominent concentric,predominantly mid-apical hypertrophy, alongside a mild pericardial effusion (*Summary Figure, panel B2*). Global longitudinal strain was reduced at −6.2% with a heterogeneous strain map (*Figure 1B*). The left ventricle (LV) size and ejection fraction (EF) were normal. Diastolic dysfunction with elevated filling pressures was noted (septal *E*/*e′* 15, left atrial volume index 53 mL/m^2^). There was bi-atrial dilatation. The right ventricle (RV) was normal in size with free wall hypertrophy (maximum 11 mm), normal radial, and impaired longitudinal systolic function. There was mild mitral regurgitation and a small pericardial collection (maximum 9 mm). This was a significant change from the echocardiogram performed 10 years prior, which had only shown mild focal apical hypertrophy, no pericardial effusion, and no systolic or diastolic dysfunction. Strain imaging was not part of the protocol at the time. Up until the 2023 scan, interval imaging remained largely stable (see *Table 2*).

His exercise tolerance test (ETT) was terminated due to symptoms after 12 min of the Bruce protocol after reaching 90% of the predicted maximum heart rate (HR). There were some isolated ventricular ectopic beats on recovery. This was a significant decline compared to a previous ETT performed 3 years earlier, where he was able to complete 21 min reaching 98% of his age-related predicted maximum HR.

Cardiovascular magnetic resonance (CMR) revealed a normal LV size with loss of longitudinal function but preserved radial function (EF 69%). There was significant global LV hypertrophy more prominent within the mid-apical segments (*Summary Figure, panel B3*). The RV was hypertrophied, and the size was normal with reduced longitudinal function but preserved radial function (EF 64%). Late gadolinium imaging revealed extensive subendocardial myocardial enhancement more evident in the mid-to-apical segments (*Summary Figure, panel B4*) and diffuse myocardial enhancement of mid-to-apical septum and anterior walls. There was also enhancement in the RV, atrial walls, and valves. Native T1 mapping and extracellular volume values were elevated. A circumferential pericardial effusion with a maximum dimension of 13 mm was confirmed. These changes were progressive compared to a CMR performed 18 months earlier, which had showed apical hypertrophy with a wall thickness of 11 mm in the apical segments associated with mild diffuse fibrosis and no pericardial effusion (*Summary Figure, panel A3*). The interval change felt somewhat suspicious for the patient’s mild and so far relatively static HCM phenotype, and further investigations were undertaken. The possibility of cardiac amyloidosis was raised given the overall picture and taking into consideration the comparison of ECGs, echocardiograms, and CMR appearance. The presence of the pericardial effusion would have also been unusual for a relatively stable HCM phenotype.

A monoclonal paraproteinaemia was demonstrated on serum electrophoresis. Bone marrow biopsy revealed a clonal population of plasma cells with a 5% burden with the following phenotype: CD19−, CD27+, CD20−, CD117−, and heterogeneous CD56 expression. An AL cardiac amyloidosis^3^ secondary to multiple myeloma co-existing with apical HCM was diagnosed. Whilst bone marrow biopsy was negative, endomyocardial biopsy confirmed AL amyloid infiltration. The patient was referred to the haemato-oncology multidisciplinary team for assessment and initiation of urgent treatment. This is currently ongoing as part of a clinical trial. His myeloma was staged as R-ISS Stage I (Revised Multiple Myeloma International Staging System^4^), and no high-risk FISH^5^ abnormality was detected. He remains breathless on exertion and is managed from a heart failure point of view with bumetanide 1 mg daily, dapagliflozin 5 mg daily, and a 1.5 L fluid restriction.

## Discussion

This rare case is a reminder to consider acquired pathology in patients followed up for cardiomyopathies. It illustrates the need for early detection and clinical vigilance; delay to diagnosis of amyloid may impact prognosis. Symptoms in excess of morphological findings should prompt the evaluation of alternative pathology in case of patients followed up for mild chronic cardiomyopathies. Multimodality imaging is key in the evaluation and follow-up of cardiomyopathy. The 2023 ESC guidelines for the management of cardiomyopathies give a Class I recommendation to follow-up clinically stable patients with cardiomyopathy using ECG and echocardiography every 1–2 years.^6^ If there is a change in clinical status, a Class I recommendation is given to offer multimodality imaging. The 2020 ESC Sports Cardiology guidelines further refine this, recommending annual follow-up for patients who exercise on a regular basis.^7^ The level of evidence behind all these recommendations is C underlining the need for further research in the area. Nonetheless, our case illustrates the benefit of adhering to those recommendations.

Prior to developing cardiac amyloid, the patient was an avid athlete. In the absence of high-risk features, the ESC guidelines supported this with a Class IIb recommendation for participation in high-intensity and competitive sports. The symptomatic burden of AL amyloid is significant, and the patient is likely to find high-intensity exercise intolerable, compounded by side effects of chemotherapy.

The patient remains under cardiology follow-up, and an interval CMR scan is pending to assess the response to treatment in terms of possible regression in LGE, native T1, and ECV, as evidenced by limited literature.

In summary, this is an unusual case of a middle-aged endurance athlete with known apical HCM and a new diagnosis of cardiac AL amyloid, which underscores the importance clinical vigilance, multimodality imaging, and longitudinal surveillance in patients with known cardiomyopathies.

## Lead author biography


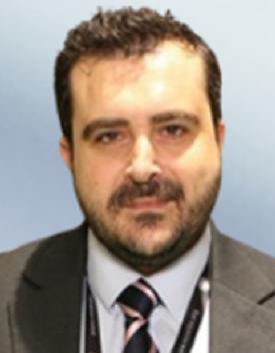


Dr Emmanuel Androulakis is a consultant cardiologist at Kings College Hospital NHS Foundation Trust (London, UK) with a specialist interest in cardiac imaging and inherited cardiac conditions.

## Data Availability

The data underlying this article will be shared on reasonable request to the corresponding author.
